# Mechanosensitivity of nicotinic receptors

**DOI:** 10.1007/s00424-012-1132-9

**Published:** 2012-06-26

**Authors:** N. Clara Pan, Jin Jin Ma, H. Benjamin Peng

**Affiliations:** 1 Division of Life Science, State Key Laboratory of Molecular Neuroscience, The Hong Kong University of Science and Technology, Clear Water Bay, Kowloon, Hong Kong; 2 Present Address: Department of Neurobiology, Beijing Institute for Neuroscience, The Capital Medical University, Beijing, China 100069

**Keywords:** Acetylcholine receptor, Mechanosensitivity, Rapsyn, Neuromuscular junction

## Abstract

Nicotinic acetylcholine receptors (nAChRs) are heteropentameric ligand-gated ion channels that mediate excitatory neurotransmission at the neuromuscular junction (NMJ) and other peripheral and central synapses. At the NMJ, acetylcholine receptors (AChRs) are constantly exposed to mechanical stress resulting from muscle contraction. It is therefore of interest to understand if their function is influenced by mechanical stimuli. In this study, patch-clamp recordings showed that AChR channel activity was enhanced upon membrane stretching in both cultured *Xenopus* muscle cells and C2C12 myotubes. To examine how this property is physiologically regulated, effects of membrane-intrinsic and membrane-extrinsic factors on AChRs expressed in HEK293T cells were studied. As in muscle cells, AChR single channel currents recorded under cell-attached configuration were significantly increased—without change in current amplitude—when negative pressure was applied through the patch pipette. GsMTx-4, a peptide toxin that blocks mechanically activated cation channels, inhibited this effect on AChRs. The mechanosensitivity decreased when cells were treated with MβCD, latrunculin A or cytochalasin D, but increased when exposed to lysophosphatidylcholine, indicating contributions from both membrane lipids and the cytoskeleton. Rapsyn, which binds to AChRs and mediates their cytoskeletal interaction in muscle, suppressed AChR mechanosensitivity when co-expressed in HEK293T cells, but this influence of rapsyn was impaired following the deletion of rapsyn’s AChR-binding domain or upon cytoskeletal disruption by cytochalasin D. These results suggest a mechanism for regulating AChR’s mechanosensitivity through its cytoskeletal linkage via rapsyn, which may serve to protect the receptors and sarcolemmal integrity under high mechanical stress encountered by the NMJ.

**Electronic supplementary material:**

The online version of this article (doi:10.1007/s00424-012-1132-9) contains supplementary material, which is available to authorized users.

## Introduction

Ion channels are essential regulators of cellular functions. They are broadly categorized as voltage- or ligand-gated, depending on whether they are activated by membrane potential changes or by ligand binding. In addition, mechanosensitive channels (MSCs) activated or inhibited by membrane stretch are widely distributed in many types of cells [2, 22, 24]. MSCs mediate a variety of functions, ranging from the highly specialized mechanical transduction mediated by channels at the tip of stereocilia in vertebrate hair cells [20, 40, 48], to the general maintenance of membrane integrity as in the case of MscL/S channels in the prokaryotic membrane [24]. MSCs are also abundantly expressed in skeletal muscle [13, 14, 19] where they are involved in the maintenance of sarcolemmal integrity; their increased activity in dystrophic muscle is correlated with muscle pathology.

Also highly expressed in vertebrate skeletal muscle are the ligand-gated nicotinic acetylcholine receptors (AChRs) that mediate synaptic transmission at the neuromuscular junction (NMJ). Each AChR is a pentamer composed of two α subunits and one each of β , γ and δ subunits in embryonic muscle, with γ being replaced by an ε subunit in the adult [29]. The five subunits together form a central pore for cation permeation upon ligand binding to the α subunits. At the postsynaptic membrane, AChRs are clustered to a density of ~10,000 per μm^2^ [12]. This AChR-centered postsynaptic apparatus is maintained throughout life and is essential for efficacious excitation–contraction coupling at the NMJ. In patients suffering from the acquired autoimmune disease myasthenia gravis, the high AChR site density is impaired as a result of auto-antibody-induced increase in AChR turnover and degradation [27]. AChR subunit mutations, on the other hand, cause congenital myasthenic syndrome [11]. Thus, the stability and function of AChRs are important for the NMJ’s integrity.

Being membrane-intrinsic molecules, AChRs, like MSCs, are subject to mechanical stress arising from muscular contraction. It is thus of interest to understand how AChRs cope with this stress to perform their physiological functions reliably and also if and how their gating properties are affected by shear force applied to the membrane. AChRs are aggregated and stabilized at the NMJ via a cytoskeleton-dependent mechanism which is mediated by rapsyn, a 43-kD protein that associates with AChR subunits [4] and whose targeted deletion abolishes AChR clustering in muscle [16]. Therefore, rapsyn may also be a player in AChR’s response to mechanical stress. In this study, we examined AChR’s mechanosensitivity in its natural muscle cell environment and in heterologous cells expressing this receptor as an exogenous protein. We moreover tested the influence of rapsyn on this property. We report that mechanical stretching of the membrane enhanced ligand activation of AChRs and that rapsyn suppressed AChR’s mechanosensitivity.

## Methods

### Ethical approval

The use of animals in this study was approved by the Animal Ethics Committee of the Hong Kong University of Science and Technology.

### Cell cultures

Myotomal muscle cells were cultured from *Xenopus* embryos as previously described [34]. Electrophysiological experiments were performed in cultures 1–2 days old. HEK293T cells were cultured in DMEM supplemented with 10 % fetal bovine serum (FBS) and incubated at 37 °C in 5 % CO_2_, and 1 day before transfection, the cells were transferred to 24-well plates. Transfection was carried out with cDNAs encoding AChR’s four subunits and green fluorescent protein (GFP) at the ratio α/β/γ/δ/GFP = 0.6:0.3:0.3:0.3:0.1 (micrograms) per well. For co-transfection of AChR and rapsyn, 0.3 μg rapsyn–GFP cDNA per well was additionally included. At 90 % confluence, cells were transiently transfected using Lipofectamine2000 (Invitrogen, Carlsbad, CA). Electrophysiological experiments were performed 1–2 days after transfection. Cells from mouse C2C12 skeletal muscle line (obtained from American Type Culture Collection, Manassas, VA) were cultured in DMEM supplemented with 20 % FBS at 37 °C in 5 % CO_2_. Myoblasts were induced to differentiate into myotubes by changing the medium to DMEM containing 2 % horse serum. Electrophysiological experiments were performed on myotubes 4–5 days after changing to this differentiation medium.

### cDNAs and other reagents

Constructs encoding mouse AChR α, β, γ and δ subunits were generously provided by Dr. Stanley C. Froehner (University of Washington, Seattle). AChR ε subunit cDNA was a kind gift from Dr. Veit Witzemann (Max Planck Institute for Medical Research, Heidelberg, Germany) and rapsyn cDNA kindly provided by Dr. Jean Cartaud (Institut Jacques Monod, Universités Paris). A rapsyn construct with deletion of the coiled coil domain (Δ297–331) was generated by PCR. Both wild-type and mutant rapsyn were tagged at the C-terminus with enhanced GFP (EGFP) by inserting them into pEGFP-N1 plasmid (Clonetech, Madison, WI).

The following reagents were from commercial sources: α-bungarotoxin (BTX) and latrunculin A (LtnA) from Molecular Probes/Invitrogen (Eugene, Oregon, USA), GsMTx-4 from Peptide Institute, Inc. (Osaka, Japan), and cytochalasin D, methyl-beta-cyclodextrin (MβCD), and lysophosphatidylcholine (LPC) from Sigma (St. Louis, MO, USA).

### Patch-clamp recording

In most cases, the cell-attached mode was used in single-channel recordings. All experiments were at room temperature. The recording was conducted with Axopatch 200B patch-clamp amplifier with associated software (Axon Instruments/Molecular Devices, Union City, CA). Currents were typically digitized at 10 kHz, macroscopic records were filtered at 2 kHz. Data were analyzed with Clampfit (version 10.0; Axon Instruments) and Sigmaplot (Systat Software, Inc., Chicago, IL) software. All data are presented as mean ± SEM.

For recording from *Xenopus* muscle cells, Ringer solution (110 mM NaCl, 1 mM KCl, 1.8 mM CaCl_2_, and 8 mM HEPES, pH 7.4) was used in the pipette and bath. For recording from mammalian cells (C2C12 myotubes and HEK293T cells), the pipette solution contained 150 mM KCl, 2.5 mM MgCl_2_, 0.2 mM EGTA, and 10 mM HEPES, pH 7.4, and the bath solution contained 150 mM NaCl, 5 mM KCl, 2 mM CaCl_2_, 1 mM MgCl_2_, 10 mM glucose, and 10 mM HEPES, pH 7.4. For inside-out patch-clamp experiments on HEK293T cells, the first solution containing high KCl was used in the pipette and in the bath.

To stretch the membrane patch while recording, negative pressure was manually applied with a 1-mL syringe attached to the pipette via silicone tubing and a three-way stopcock. Pressures were monitored with a pressure gauge (PM01R, World Precision Instruments, Sarasota, FL) and displayed by Axon pClamp10 software. The pressure trace is shown in figures together with current recordings with the baseline showing the atmospheric pressure (negative pressure = 0 mmHg) and downward steps showing the magnitude of the negative pressure. A scale for the negative pressure is included in each figure where current recordings are shown. The patch diameter was approximately 1 μm. The value of NPo was calculated by using “single-channel search” function in the Clampfit software. For data obtained from the same cell, paired *t* test was used whereas unpaired *t* test was adopted for analyzing data collected from a group of cells.

## Results

### Mechanosensitivity of AChRs in cultured skeletal muscle cells

**Fig. 1:**
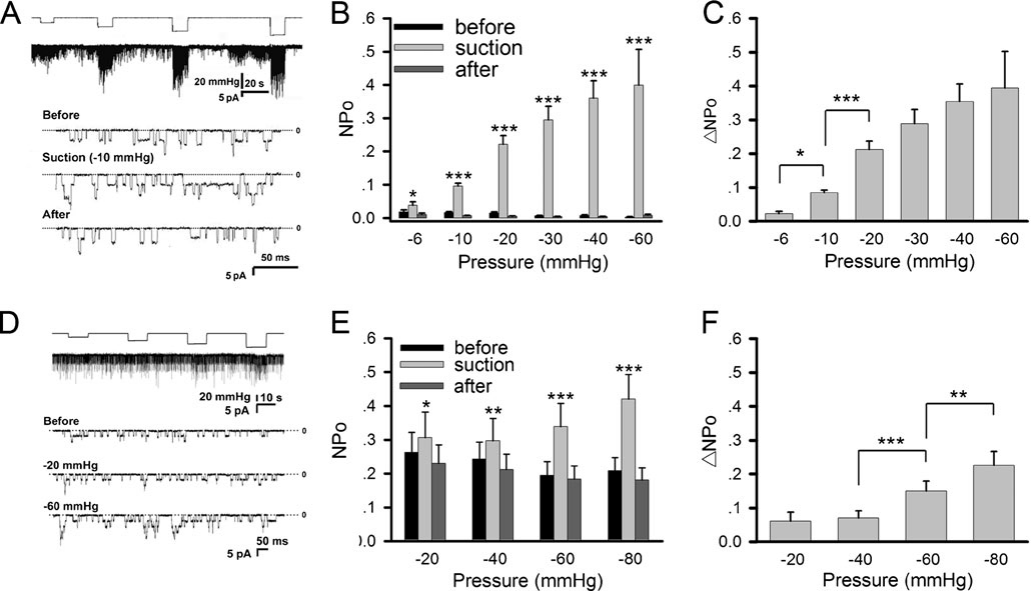
Mechanosensitivity of AChRs in muscle cells. AChR activity was studied in cultured *Xenopus* myotomal muscle cells **(a–c)** and C2C12 myotubes **(d–f)**. **a** Sample traces of ACh-induced single-channel currents from *Xenopus* muscle cells recorded with the patch-clamp method under negative pipette pressure of different magnitudes. Pipette ACh concentration = 0.2 μM, holding potential = +70 mV, cell-attached mode. **b** Channel activity NPo and **c** its difference ΔNPo under different negative pressures. For the NPo plot, values before (*black*), during (*gray*), and after (*dark gray*) negative pressure application are shown. Data are mean ± SEM, number of patches *n* = 9, 91, 46, 46, 44, 18 for negative pressures of −6, −10, −20, −30, −40, −60 mmHg, respectively. **d** AChR single channel currents recorded from C2C12 myotubes under different negative pressures. ACh concentration = 0.5 μM. **e** NPo values before (*black*), during (*gray*), and after (*dark gray*) negative pressure application. **f** ΔNPo data from C2C12 cells. Data from 20 patches at each negative pressure were pooled. Statistics: *p < 0.05; **p < 0.01; ***p < 0.001 (Student’s paired *t* test). For NPo plots, comparisons were made between values obtained during and before negative pressure application

Acetylcholine-induced single-channel currents were recorded from cultured *Xenopus* myotomal muscle cells using the cell-attached patch-clamp technique. To study the mechanosensitive property of these receptor channels, the membrane patch under recording was stretched by negative pressure applied through the pipette by suction. The duration of the negative pressure application was about 10 s for each step. After each application, the negative pressure was released to allow cell recovery before the next step was applied. Membrane stretching caused by negative pressure as low as −10 mmHg resulted in a striking increase in AChR single channel opening activated by 200 nM ACh in the pipette (Fig. 1a). This was accompanied by an increase in the occurrence of multiple channel activation and prolongation of the mean open time. Channel opening returned to normal state after the negative pressure was released. To quantify this effect, we calculated the channel activity, NPo (Fig. 1b), and the difference before and under negative pressure application, ΔNPo (Fig. 1c). In a strength-dependent and reversible manner, negative pressure enhanced ligand-activated AChR opening.

In these muscle cells, single channel currents displayed two distinct amplitudes of 6 and 8 pA, corresponding to AChRs containing γ and ε subunits, respectively [26]. The resting membrane potential was about −70 mV. Together with the pipette holding potential of +70 mV, a calculated net membrane potential of −140 mV was imposed on the patch membrane. This allowed us to deduce the single-channel conductance of γ- and ε-containing AChRs at 43 and 57 pS, respectively. Although membrane stretching increased channel activity, the AChR single channel currents were not affected (Fig. 1a; described in more detail below).

To determine if this mechanosensitive property was unique to AChRs in *Xenopus* muscle cells, a similar study was carried out on C2C12 myotubes. AChRs in these cells also exhibited mechanosensitivity in response to membrane stretching by negative pressure applied through the patch-clamp pipette (Fig. 1d–f). This increase in channel opening was not as large as that in *Xenopus* primary muscle cultures. These mechanosensitive, ACh-activated channels were identified unambiguously as nicotinic receptors by the finding that their currents were abolished by the nicotinic antagonist α-bungarotoxin (Supplemental Fig. S1a).

### AChR mechanosensitivity in heterologous cells

**Fig. 2:**
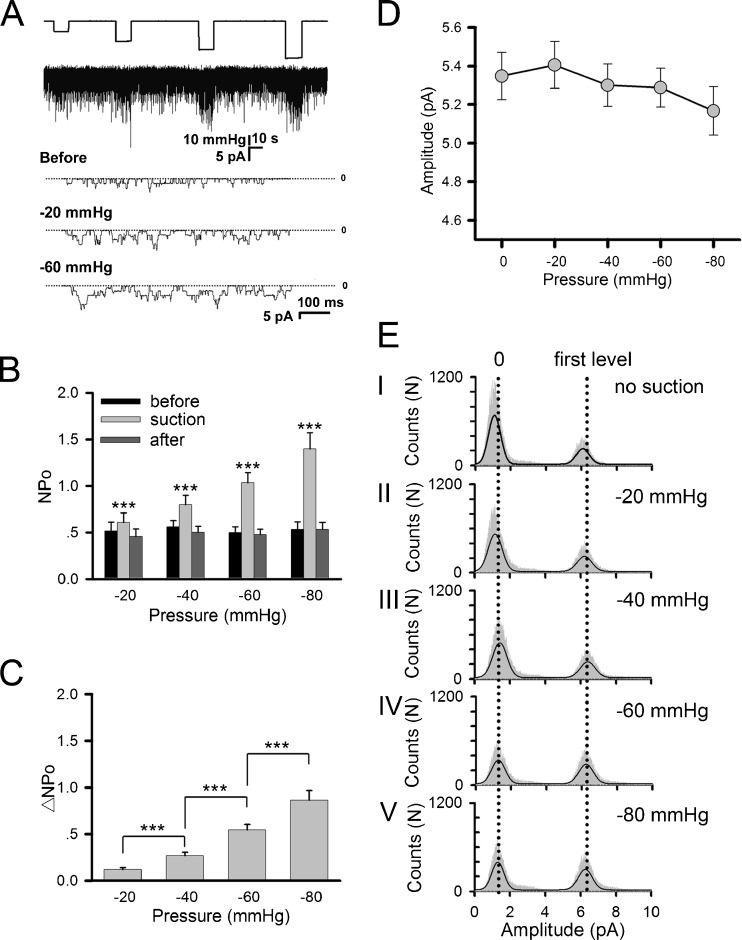
Mechanosensitivity of AChRs exogenously expressed in HEK293T cells. Cells were transfected with AChR subunits α, β, δ, and γ. **a** Sample traces of ACh-induced currents under different negative pressures. Included in the pipette was 0.5 μM ACh. **b** Channel activity NPo and **c** its difference ΔNPo under different negative pressure levels; the former shows values before (*black*), during (*gray*), and after (*dark gray*) negative pressure application; *n* = 26, 24, 22, 10 patches for −20, −40, −60, −80 mmHg, respectively. *Symbols* denoting statistical significance are the same as in Fig. 1. **d** Mean amplitudes of single channel currents at different negative pressures. These values were calculated from all-points amplitude histograms as shown in (**e**), taking the difference between the first and second peak as the mean single-channel AChR current amplitude

To further characterize the mechanosensitivity of nicotinic receptors, we introduced AChRs into HEK293T cells that do not express these receptors endogenously. Transfection of cDNAs encoding muscle-type AChR subunits α, β, γ, and δ into these cells led to the expression of AChRs at the cell surface, as shown by the appearance of ACh-induced currents (Fig. 2a and Supplemental Fig. S1b). The mechanosensitivity of these exogenous AChRs was readily apparent (Figs. 2a, b), and at negative pressures over −60 mmHg, membrane stretching more than doubled the channel opening with respect to control (Fig. 2c). However, while channel activity was dramatically enhanced, the amplitude of single-channel current was not altered (Fig. 2d). This is also shown in the histogram of single channel currents in Fig. 2e. The net ACh-induced current amplitude after baseline subtraction was about 5.1 pA for all negative pressure levels tested (Fig. 2e). In these experiments, a pipette holding potential of +70 mV was used in all recordings. Since the resting potential of HEK293 cells was at −40 mV [44], the calculated conductance of AChR single channel was ~46 pS under the driving force of −110 mV net membrane potential. This value was consistent with that of muscle γ-containing AChRs as described previously.

**Fig. 3:**
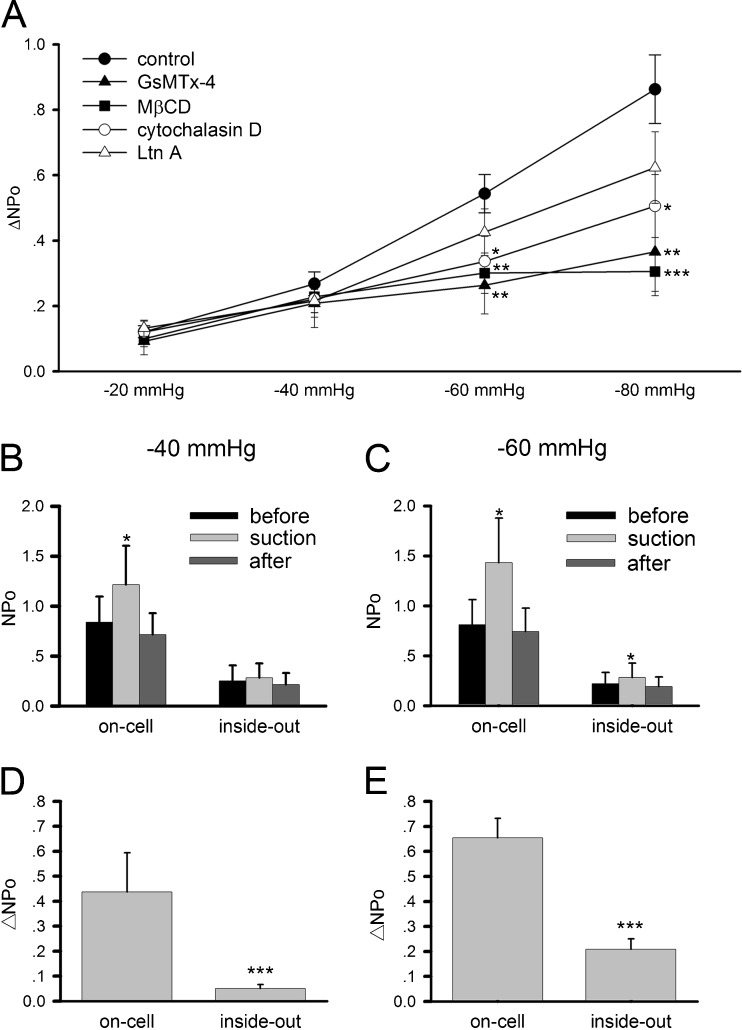
Analyses of AChR mechanosensitivity in HEK293T cells. **a** Effects of different pharmacological agents on ΔNPo. Cell-attached single-channel recording was conducted. GsMTx-4 (1 μM), a mechanosensitive channel blocker, was included in the recording pipette. Cells were incubated before recordings for 20 min in 10 mM MβCD, a cholesterol-depleting agent; 30 min in 2 μM cytochalasin D, an F-actin inhibitor; or for 2 h in 5 μM latrunculin A, another F-actin inhibitor. Number of patches recorded: 26 (control), 26 (GsMTx-4), 19 (MβCD), 29 (cytochalasin D), and 19 (latrunculin A). **b–e** Comparison of on-cell and inside-out recordings. Data are mean ± SEM based on *n* = 10 patches (for each negative pressure). The same membrane patch was first recorded on-cell and then detached from the cell for inside-out measurement with a pipette holding potential of +70 mV and 0.5 μM ACh. *p < 0.05; **p < 0.01; ***p < 0.001

As another test to verify the mechanosensitivity of AChR channels, we used GsMTx-4, a peptide toxin isolated from the venom of the Chilean rose tarantula (*Grammostola spatulata*) that blocks stretch-activated cation channels [43]. Inclusion of this toxin at 1 μM in the ACh-containing pipette solution potently inhibited the change in AChR channel activity (ΔNPo) triggered by membrane stretching, an effect especially noticeable at high negative pressures (Fig. 3a).

As noted above, the AChR subunit makeup changes during development from the embryonic type α(2)βδγ to adult type α(2)βδε. The foregoing results from heterologous cells were obtained using the embryonic AChR subunit composition. Recording was thus also performed on HEK293T cells expressing the adult combination. Compared to γ-containing channels, the ε-containing ones have a shorter single-channel opening time and higher current amplitude. Nevertheless, they also clearly exhibited mechanosensitivity in response to negative pipette pressure (Supplemental Fig. S2).

### Effects of membrane and cytoskeletal perturbation on AChR’s mechanosensitivity

Previous studies have implicated both the membrane lipid bilayer and the cytoskeleton as determinants in ion channels’ mechanosensitivity. To understand the cell membrane’s influence on AChR’s mechanosensitivity, the membrane lipid content was perturbed by treating AChR-expressing HEK293T cells with the cholesterol-depleting agent methyl-β-cyclodextrin (MβCD) [8, 31]. Following incubation with 10 mM MβCD for 20 min, cell-attached single-channel recording showed that AChRs became much less sensitive to membrane stretch when the negative pressure was in the range of −60 to −80 mmHg (Fig. 3a). In the absence of membrane stretching, MβCD did not affect the gating of AChRs.

**Fig. 4:**
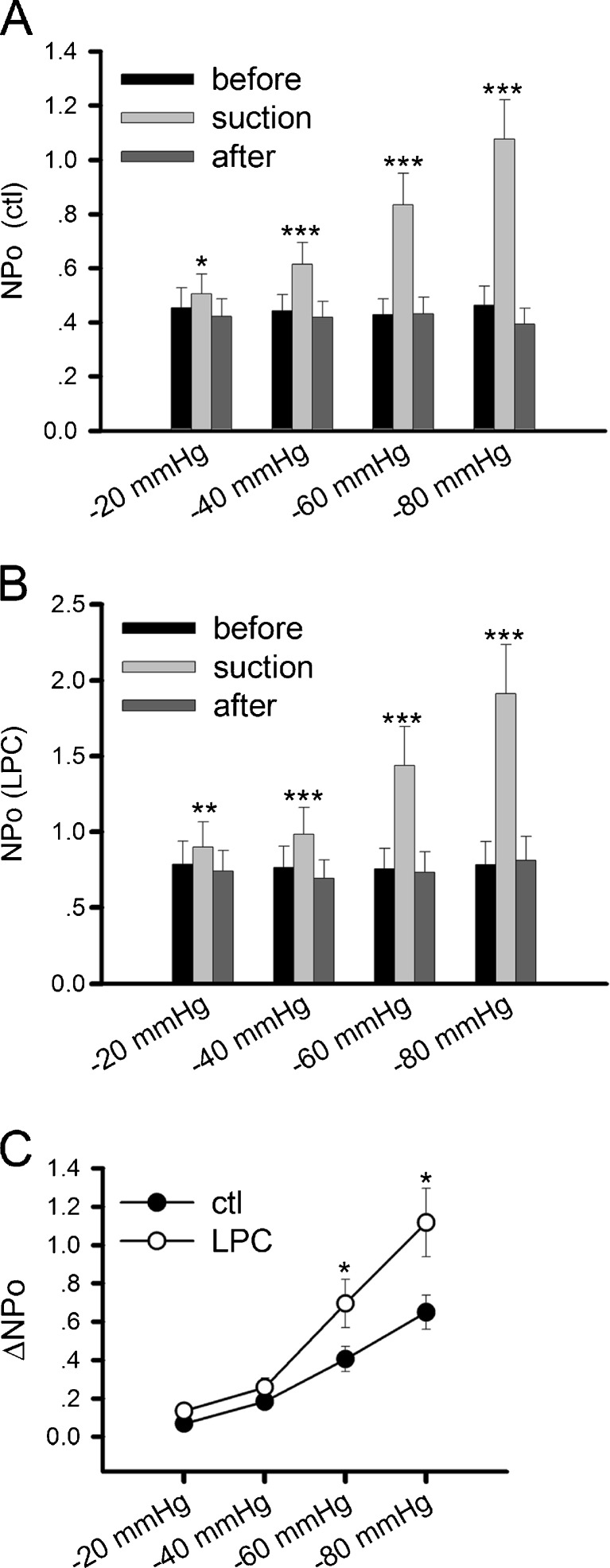
Effect of LPC on AChR mechanosensitivity. AChR-expressing HEK293 cells were pretreated with LPC for 10 min before patch-clamp recording. **a** Channel activity NPo of AChRs before (*black*), during (*gray*), and after (*dark gray*) negative pressure application in control cultures; *n* = 16 patches. **b** NPo of LPC-treated AChRs; *n* = 16 patches. **c** The difference of NPo for control and LPC-treated AChRs at different negative pressure

Previous studies have shown that the gating property of bacterial mechanosensitive channels MscL and MscS can be profoundly modified by the intercalation of the conical phospholipid lysophosphatidylcholine (LPC) [35, 46]. LPC added to one side of the lipid bilayer causes an increase in MscL’s mechanosensitivity. To further understand the involvement of the membrane environment on AChR’s mechanosensitivity, we pre-incubated receptor-expressing HEK293 cells with 5 μM LPC for 10 min according to previously published method [1] and then recorded ACh-induced single-channel currents. As shown in Fig. 4, the increase in ligand-induced channel activity due to negative pressure was significantly enhanced, especially at higher pressures, as a result of LPC application.

The action of the cytoskeleton was studied by disrupting F-actin assembly and stability with latrunculin A (LtnA) and cytochalasin D. LtnA, isolated from the Red Sea sponge *Latrunculia magnifica*, interferes with actin polymerization through binding and sequestering monomeric G-actin [9, 42]. Cytochalasin D, a fungal metabolite, binds to nuclei and filaments of F-actin and blocks the incorporation of G-actin [7]. Both agents also destabilize and depolymerize F-actin. Pre-incubation of HEK293T cells expressing AChRs with either 2 μM cytochalasin D for 30 min or 5 μM LtnA for 2 h caused significant reduction in the receptors’ sensitivity to membrane stretch at negative pressures of −60 mmHg and above (Fig. 3a). However, these actin depolymerizing agents did not affect AChR gating in the absence of membrane stretching.

The importance of the intact cytoskeleton was further probed by recording from inside-out membrane patches. In this configuration, the pipette-associated membrane patch is detached from the cell and (therefore) its underlying cytoskeleton, giving it the “inside-out” configuration. Negative pressure was applied to the isolated membrane to cause its stretching and concave distortion in the same way as in an intact cell. In sharp contrast to what was seen in cell-attached recordings, AChRs’ mechanosensitivity in inside-out patches was almost absent at −40 mmHg negative pipette pressure and weak at −60 mmHg (Fig. 3b–e). These data suggest that the mechanosensitivity of AChRs is conveyed through both the lipid bilayer and the cytoskeleton.

### The function of rapsyn in AChR mechanosensitivity

**Fig. 5:**
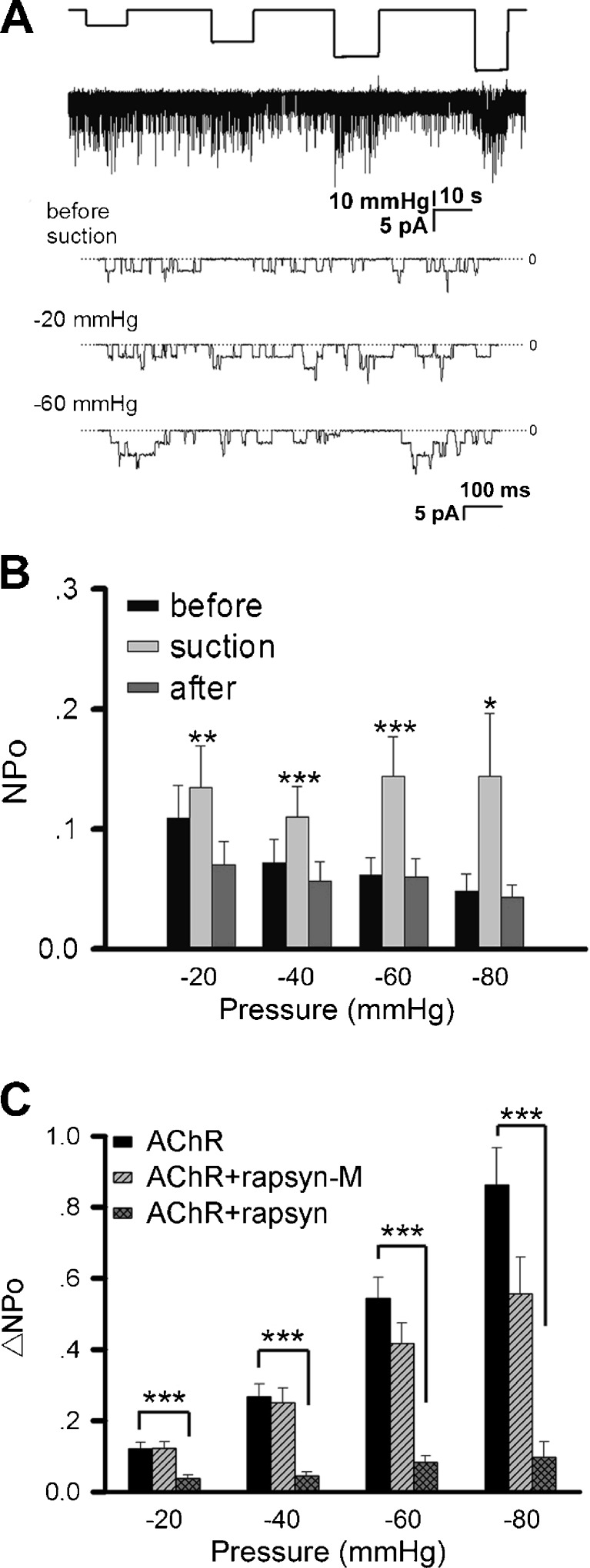
The effect of rapsyn on AChR mechanosensitivity. HEK293T cells were transfected with cDNAs encoding AChR subunits plus one encoding rapsyn. **a** Sample current recording of AChRs under different pipette negative pressures in the cell-attached mode; pipette holding potential +70 mV with 0.5 μM ACh. **b** NPo of ACh-induced single-channel currents from rapsyn-co-expressing cells before (*black*), during (*gray*), and after (*dark gray*) negative pressure application. Note the reduced scale of *y*-axis compared to Figs. 1 and 2. *N* = 21, 18, 11, 6 patches for −20, −40, −60, −80 mmHg, respectively. **c** ΔNPo values of AChRs expressed alone, with rapsyn or with mutant rapsyn lacking the AChR-binding domain (rapsyn-M). Data from 18 to 26 patches were pooled. *p < 0.05; **p < 0.01; ***p < 0.001

By comparing data from *Xenopus* and C2C12 muscle cells (Fig. 1b, c, e, f) with those from HEK293T cells (Fig. 2b, c), one can see that the mechanosensitivity of endogenous AChRs in muscle was less than that of exogenous ones expressed in heterologous cells. A major difference between these cell types that could have a bearing on AChRs is the association of AChRs with rapsyn, which is an AChR-binding protein expressed mainly by muscle cells [4]. Rapsyn is essential for AChR clustering at the NMJ and is generally thought to mediate the receptor’s interaction with the cytoskeleton. To examine a potential role of rapsyn in regulating AChR’s mechanosensitivity, it was co-expressed with the receptor subunits in HEK293T cells. As shown in Fig. 5a, b, membrane stretching through pipette negative pressure still increased AChR activity. However, this increment was significantly less than that shown by receptors expressed without rapsyn (Fig. 2b; note the reduced scale of *y*-axis in Fig. 5b relative to Figs. 1 and 2). This change becomes clearer when ΔNPo values are compared: rapsyn potently suppressed ΔNPo throughout the range of negative pressures tested (Fig. 5c).

Additional experiments showed the specific effect of rapsyn’s association with AChR on the channel’s mechanosensitivity. Rapsyn interacts with AChRs via its coiled coil domain [37, 38]. Deletion of this domain (in the mutant rapsyn-M) dramatically reduced rapsyn’s inhibitory effect on AChR’s mechanosensitivity (Fig. 5c), suggesting that rapsyn binding to AChR effectively suppresses the influence of mechanical stimuli on the receptor’s activity.

**Fig. 6:**
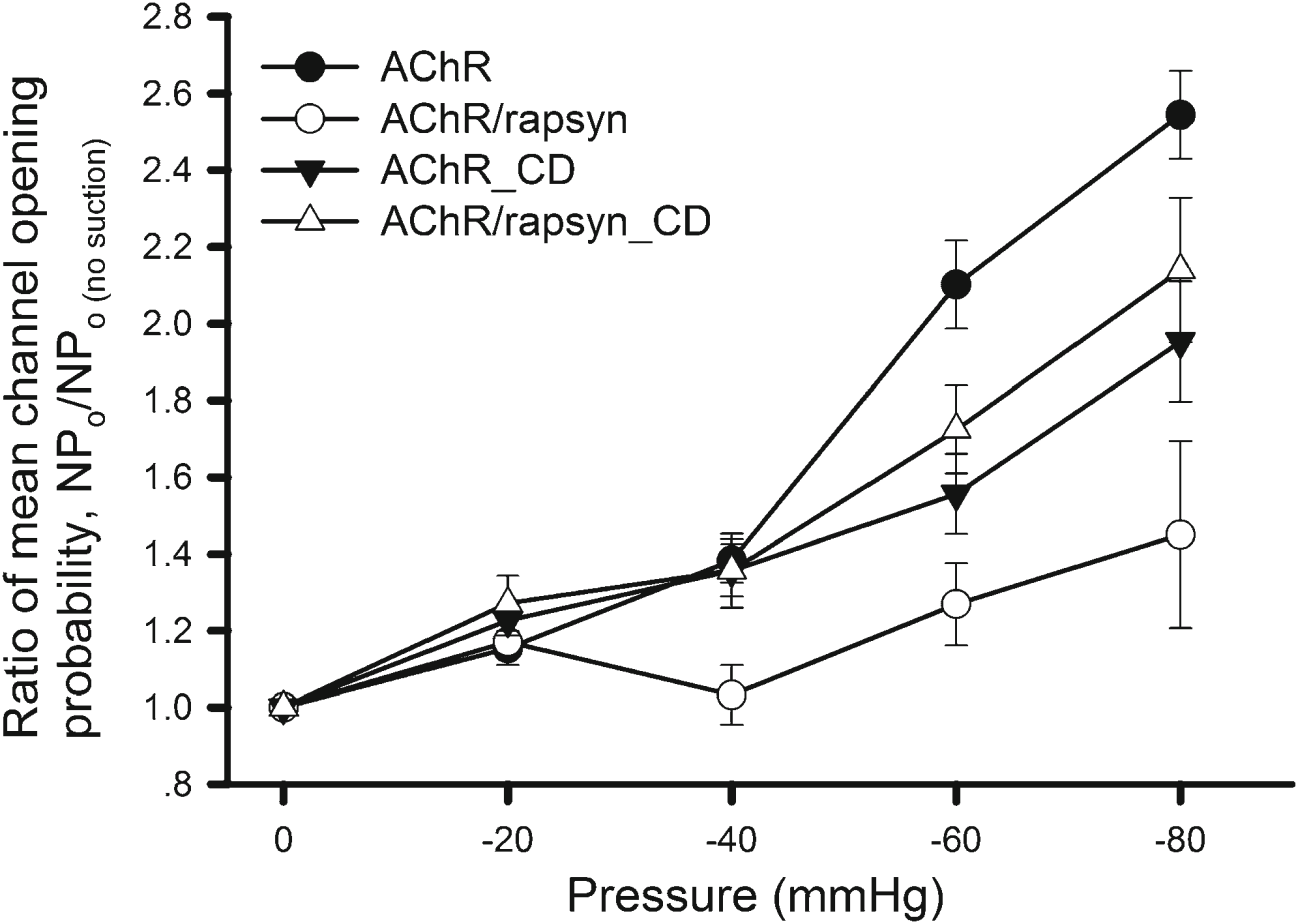
Ratio of NPo under different experimental conditions. HEK293T cells expressing AChRs alone or together with rapsyn were recorded in the presence or absence of cytochalasin D (*CD*; 2 μM, 30 min pre-incubation). The NPo values obtained at each negative pressure were divided by the respective basal NPo. Data from more than 20 patches were pooled for each point. Student’s *t* test showed no significant difference between the results of AChR_CD and AChR/rapsyn_CD (*p* > 0.2)

Co-expression of wild-type rapsyn leads to AChR cluster formation in heterologous cells [15, 36], which was also observed in this study (NCP and HBP, unpublished observations). This clustering lowered the density of diffuse AChRs at the cell surface, as shown by current recording under whole-cell mode (Supplemental Fig. S3). Because of this reduction in diffuse AChR site density, we also plotted the ratios of NPo under each negative pressure to that under normal pressure (before suction) and normalized them (Fig. 6). By calculating this NPo ratio, we can rule out the possibility that reduction in channel activity (NPo) is due to a decrease in surface AChR number (N). As with the ΔNPo plot in Fig. 5c, here again rapsyn’s effect on reducing AChR’s mechanosensitivity was obvious (Fig. 6) even though rapsyn expression also lowered the density of diffuse receptors.

To further understand the interplay between rapsyn and the cytoskeleton in mediating the mechanosensitivity of AChRs, the effect of cytochalasin D on AChR and rapsyn co-expressing cells was studied. Cytochalasin D treatment reduced AChR’s mechanosensitivity in the absence of rapsyn (Fig. 3a). However, in rapsyn co-expressing cells, cytoskeletal disruption caused an increase in AChR opening induced by membrane stretch, although the level was still lower than that seen in cells expressing AChRs alone (Fig. 6). Similar effects of cytochalasin D were also observed in C2C12 cells which endogenously express rapsyn (Supplemental Fig. S4). These data suggest that rapsyn, through its binding to AChRs, suppresses the receptor’s mechanosensitivity. And though the exact molecular mechanism of this suppression is not known, the integrity of the cortical F-actin cytoskeleton appears to be critical for rapsyn’s ability to perform this function effectively.

## Discussion

In this study, the mechanosensitivity of nicotinic AChR in the skeletal muscle was characterized for the first time. This property was manifested as membrane stretch-induced increase in the channel activity (but not channel conductance) after ligand binding to the receptor. Unlike other MSCs in muscle membrane that respond to membrane stretch alone [13, 14, 19], mechanical stimulus only modulated ligand-activated AChR opening. Mechanosensitivity was exhibited by embryonic (α_2_βδγ) as well as adult (α_2_βδε) AChRs. Since this property was manifested by endogenous AChRs in muscle cells and exogenous ones expressed in HEK293T, the mechanosensitivity is a general property of this channel and not unique to its muscle membrane environment.

AChR’s mechanosensitivity resembles that of another ligand-gated channel, the NMDA receptor. Membrane stretching has been reported to increase the NMDA response of cultured embryonic mouse neurons [32], where mechanical force only modulates ligand-induced activation and does not by itself cause channel opening, much as with AChRs here. The mechanosensitive property seems unique to NMDA receptors, for it was not observed among other receptors in mouse neurons, including kainate, glycine, and GABA receptors [32]. In another study, purified NR1a and NR2A subunits of the NMDA receptor were reconstituted into liposomes, and it was found that only the lipid bilayer was involved in the mechanosensitivity [23]. For the AChR, both membrane lipids and the cytoskeleton were found to be involved in generating its mechanosensitivity through studies conducted in HEK293T cells as reported here. Interestingly, the co-expression of the receptor-associated protein rapsyn can counteract the effect of the mechanical force.

AChR’s response to mechanical stimuli, like that of other MSCs, was blocked by the spider toxin GsMTx-4 [6] and was detected in both *Xenopus* and mammalian (C2C12) muscle cells in this study. The mechanosensitivity of C2C12 receptors was smaller than their *Xenopus* primary muscle counterparts. AChR’s channel open time in chick myotubes, however, is apparently not affected by membrane stretching [19]. Thus, there could be a species-dependent difference in AChR mechanosensitivity. Moreover, in preliminary studies using cortical neurons isolated from E18 rat brain, we recorded no significant enhancement of ACh single-channel currents upon applying negative pressure through the patch-clamp pipette (Supplemental Fig. S5). We also failed to observe mechanosensitivity when α7-nicotinic receptors were expressed in HEK293T cells together with the *ric3* gene (to increase the surface expression and assembly of this receptor in heterologous cells) [49, 50] (NCP and HBP, unpublished observations). Thus, for nicotinic receptors, mechanosensitivity appears to be specific to the skeletal muscle type. Interestingly, AChR’s mechanosensitivity was not dependent specifically on its native environment in *Xenopus* myotomal muscle cells in primary culture or in C2C12 myotubes; it was also observed when receptors were heterologously expressed in HEK293T cells.

In the muscle, the membrane is an important mediator of force transmission from sarcomeres to the myotendinous junction during contractile activity [33, 45]. Although we do not know the membrane tension generated during muscular activity, previous studies have shown that the intramuscular fluid pressure (IMP) reaches 270 mmHg in the soleus muscle in humans while running and under prolonged submaximal contraction, IMP can reach a level of ~570 mmHg [3, 41]. The mechanical tension generated at the cell membrane of the skeletal muscle can be expected to have a significant impact on normal synaptic transmission at the NMJ, where most AChRs are concentrated. Activated AChRs allow the non-specific passage of monovalent and divalent cations, including calcium. The enhanced channel opening caused by membrane tension could thus increase calcium ion influx at the endplate. This may lead to deleterious consequences due to calcium-mediated myopathy at the NMJ that bears similarity to slow-channel myasthenic syndrome [10, 25, 47]. In this regard, rapsyn’s restriction of AChR mechanosensitivity in muscle could be a built-in mechanism for protecting the integrity of the NMJ and the muscle fiber.

Rapsyn associates with the cytoplasmic domain of AChR subunits and is essential for the assembly of the receptors into high-density postsynaptic clusters [5, 28]. During development, the ratio of rapsyn to AChR increases, and this has been shown to contribute to the stability of AChRs at the endplate [17, 18]. Our current study suggests that this stabilization is accomplished in part through rapsyn’s ability to lower AChR’s mechanosensitivity. Thus, in addition to its well-known role in AChR clustering, a new function of rapsyn is now described—of counteracting the intrinsic AChR property of enhanced opening under membrane tension. Intriguingly, disruption of F-actin compromised the resistance of AChRs to mechanical force conferred by rapsyn co-expression. Thus, rapsyn bound to cytoplasmic domains of AChR subunits may stabilize the receptor by consolidating its interaction with the cytoskeleton. The high-density clustering of AChRs at the postsynaptic membrane is likely an important mechanism to shield them from tension by itself as the lipid microdomain surrounding the receptors is more rigid than the surrounding area. Thus, rapsyn is involved in regulating AChRs’ mechanosensitivity from both the aspect of cluster formation and in linking individual receptors to the cytoskeleton.

**Fig. 7:**
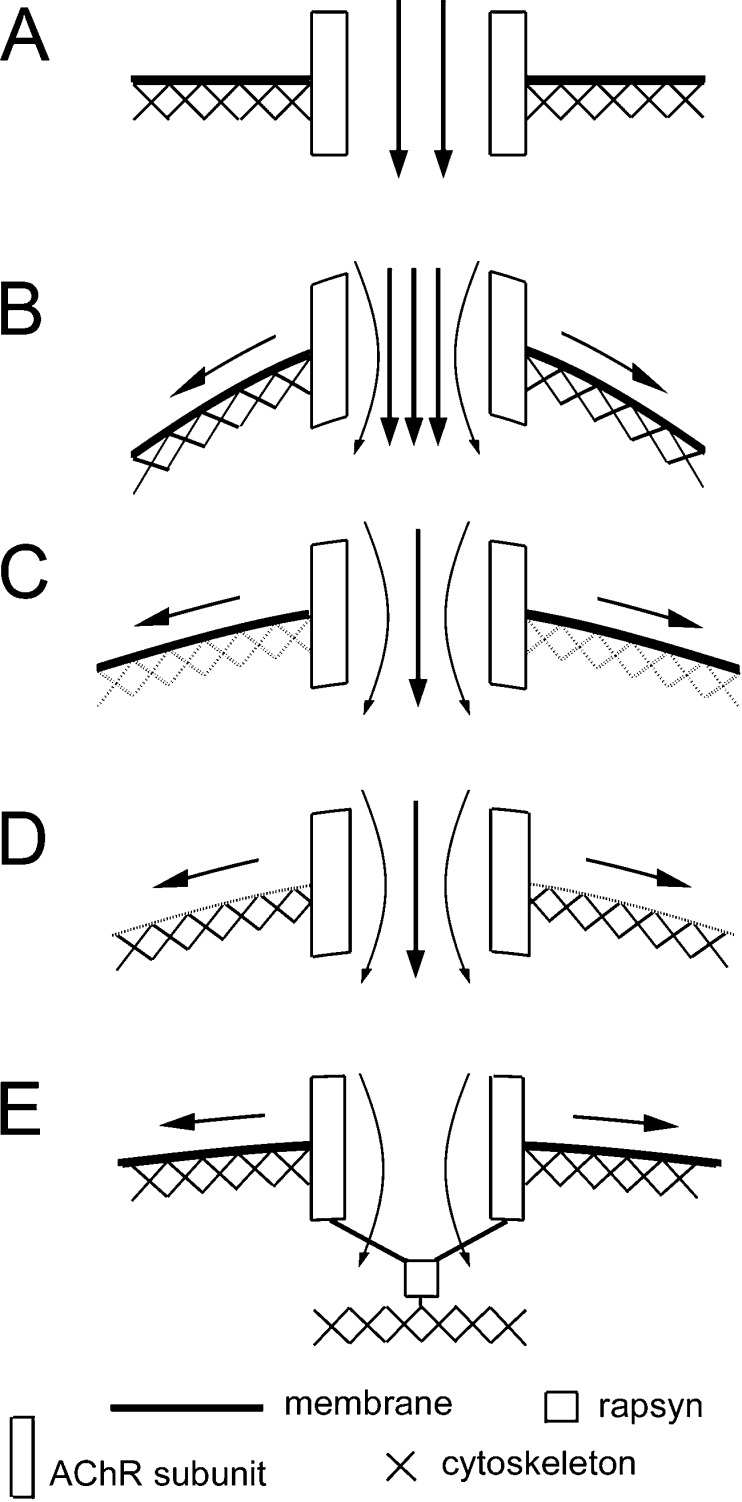
A model on the regulation of AChR mechanosensitivity by the membrane and the cytoskeleton. **a** Channel opening under zero negative pressure. As a transmembrane molecule, AChR’s gating property can potentially be influenced by the mechanical properties of the membrane and the cytoskeleton. **b** Under negative pressure application through the recording pipette, the tension generated along the plane of the membrane causes increased channel activity. **c** Disruption of the cortical F-actin cytoskeleton by latrunculin A of cytochalasin D reduces the influence of the membrane stretch force on the receptor, leading to a decrease in channel activity. **d** Membrane lipid modification that reduces its stiffness such as cholesterol depletion by MβCD also reduces the stretch force experienced by the receptor and the mechanosensitivity. **e** Rapsyn, through its interaction with AChR subunits, anchors the receptor complex to the cytoskeleton to lessen the impact of membrane stress on its gating, thus reducing the mechanosensitivity

Three broad mechanisms have been proposed to account for the stretch sensitivity of membrane ion channels, referred to as bilayer, tethered, and hybrid models [21]. The bilayer model depicts that mechanical forces are conveyed to the channel via the lipid membrane. In the tethered model, the channel is considered to be linked directly to extracellular or cytoskeletal proteins that act as a gating mechanism to mediate the effect of the mechanical force. The hybrid model combines both features. Based on studies using two F-actin disrupting agents (LtnA and cytochalasin D), we conclude that mechanosensitivity of AChRs is dependent on the intactness of the cytoskeleton. On one hand, the stretch-induced response of AChRs was suppressed by MβCD that depletes cholesterol, a known determinant in the viscoelastic property of the membrane [30, 39]. On the other hand, LPC causes an enhancement of AChR’s mechanosensitivity. This conical phospholipid causes changes in membrane curvature when intercalated into one side of the lipid bilayer, and this has been shown to increase the opening of the bacterial mechanosensitive channel [35]. Thus, our MβCD and LPC results suggest that the protein–lipid interface is also involved in AChR’s mechanosensitivity. Together, these data therefore suggest that both the cortical cytoskeleton and the membrane lipid environment participate in transmitting the tensile stress force in the plane of the membrane to AChRs to enhance ligand-induced channel opening. A model depicting the control of AChR’s mechanosensitivity is shown in Fig. 7.

The elucidation of AChR’s mechanosensitive property opens up a new way in understanding the channel’s gating mechanisms, which, coupled with AChR’s well characterized structure–function relationship, should yield further insights into this ligand-gated channel’s behavior and regulation in normal and diseased states.

## Electronic supplementary material

Below is the link to the electronic supplementary material.

Fig. S1 Characteristics of AChRs in C2C12 and HEK293T cells. **a** C2C12 myotubes. ACh-induced single-channel currents (*middle trace*) were blocked after treating cells with 1 μM BTX (*bottom trace*). **b** In non-transfected HEK293T cells, no current was elicited by 0.5 μM ACh or negative pressure applied through the pipette (*top current trace*). In cells expressing γ-subunit-containing AChRs, single channel currents were elicited by ACh (*middle trace*) and they were blocked by 1 μM BTX (*bottom trace*) (JPEG 307 kb)

Fig. S2 Mechanosensitivity of AChRs containing ε subunit. HEK293T cells were transfected with cDNAs encoding AChR α, β, δ, and ε subunits. **a** Sample current traces shown in two time scales. **b, c** NPo and ∆NPo plots of AChR single-channel currents under negative pressure of different magnitudes. Data are mean ± SEM from 16 patches. **d, e** Rapsyn co-expression reduced the mechanosensitivity of ε-containing AChRs. *p < 0.05; **p < 0.01 (JPEG 242 kb)

Fig. S3 Effect of rapsyn on AChR current density in HEK293T cells. AChRs were co-expressed with rapsyn at cDNA ratios of 1:1, 1:2, and 1:4. The current density was calculated by dividing total whole-cell current by membrane capacitance (*I*/*C*_m_). Number of cells recorded: 32 (no rapsyn), 26 (1:1), 28 (1:2), and 26 (1:4). The pipette holding potential was −70 mV. Data are mean ± SEM, **p < 0.01 (JPEG 111 kb)

Fig. S4 The effect of cytochalasin D on AChR’s mechanosensitivity in C2C12 cells. NPo of control AChR single-channel currents **(a)** and currents with cytochalasin D treatment **(b)**, before (*black*), during (*gray*), and after (*dark gray*) negative pressure application. ΔNPo comparison between control (*black*) and cytochalasin D treatment **(c)**. Number of patches *n* = 20 (control) and 21 (cytochalasin D). Myotubes were pre-incubated with 2 μM cytochalasin D for 30 min for the latter. Sample current traces in two time scales **(d, e)**. Recording pipette contained 0.5 μM ACh. Data are mean ± SEM. *p < 0.05; **p < 0.01; ***p < 0.001 (JPEG 246 kb)

Fig. S5 Response of AChRs in rat cortical neurons. **a** Dose–response of ACh-induced currents in cortical neurons under whole-cell recording mode. **b–d** Single-channel recordings of ACh-induced currents under negative pipette pressure at three ACh concentrations. In comparison to muscle nAChRs, the neuronal receptors showed negligible mechanosensitivity (JPEG 180 kb)

## Abbreviations

AChR: acetylcholine receptor
BTX: α-bungarotoxin
CD: cytochalasin D
LtnA: latrunculin A
LPC: lysophosphatidylcholine
MβCD: methyl-beta-cyclodextrin
MSC: mechanosensitive channel
NMJ: neuromuscular junction
R-BTX: rhodamine-conjugated α-bungarotoxin
