# Supplementary figures and images for: Mechanosensitivity of nicotinic receptors
Source: Pflugers Arch. 2012 Jun 26;464(2):193–203. doi: 10.1007/s00424-012-1132-9 (PMC3395360; doi:10.1007/s00424-012-1132-9)

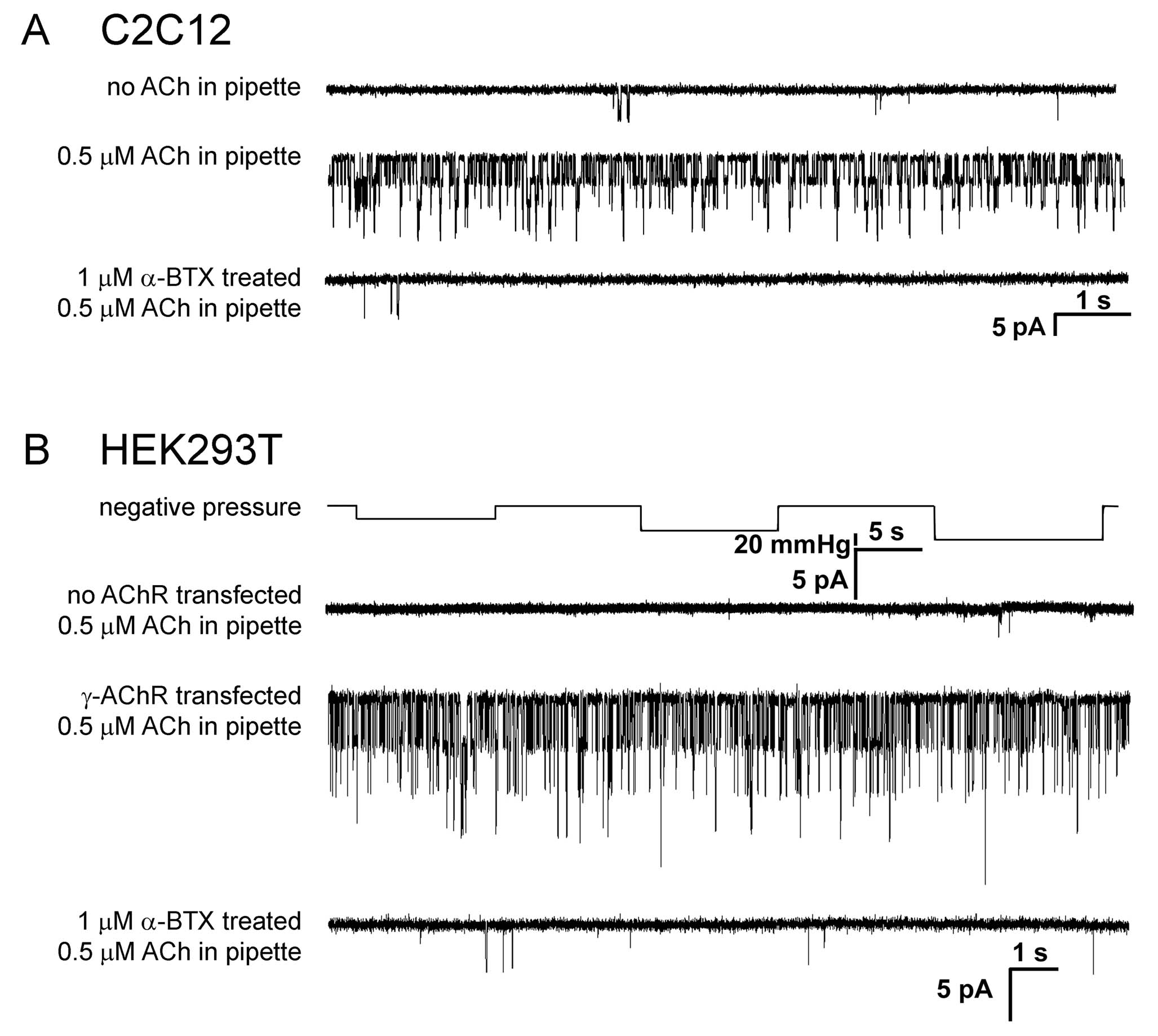

Supplement: Supplementary file 1 — Characteristics of AChRs in C2C12 and HEK293T cells. a C2C12 myotubes. ACh-induced single-channel currents (middle trace) were blocked after treating cells with 1 μM BTX (bottom trace). b In non-transfected HEK293T cells, no current was elicited by 0.5 μM ACh or negative pressure applied through the pipette (top current trace). In cells expressing γ-subunit-containing AChRs, single channel currents were elicited by ACh (middle trace) and they were blocked by 1 μM BTX (bottom trace) (JPEG 307 kb) [file 424_2012_1132_MOESM1_ESM.jpg]

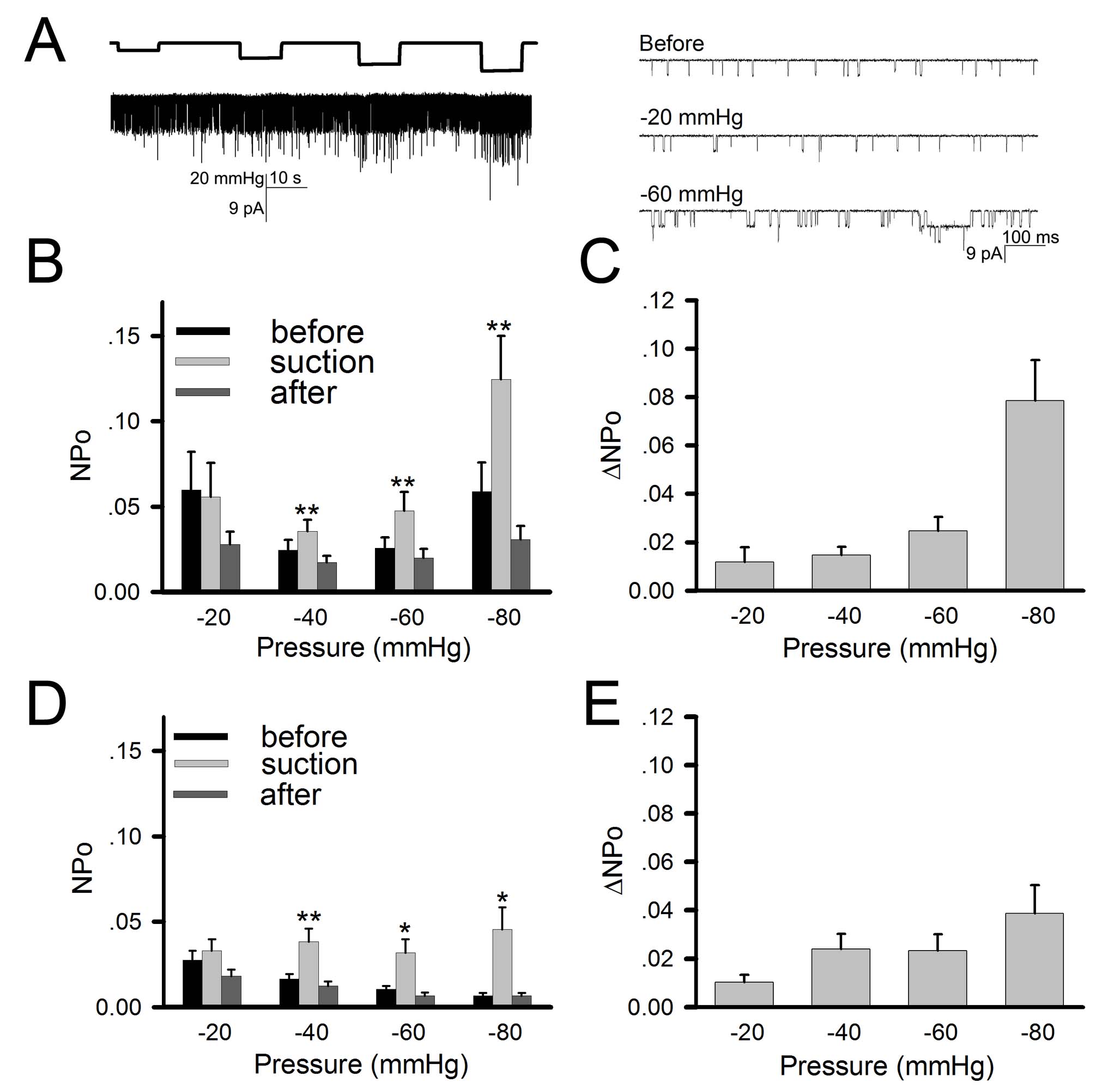

Supplement: Supplementary file 2 — Mechanosensitivity of AChRs containing ε subunit. HEK293T cells were transfected with cDNAs encoding AChR α, β, δ, and ε subunits. a Sample current traces shown in two time scales. b, c NPo and ∆NPo plots of AChR single-channel currents under negative pressure of different magnitudes. Data are mean ± SEM from 16 patches. d, e Rapsyn co-expression reduced the mechanosensitivity of ε-containing AChRs. *p < 0.05; **p < 0.01 (JPEG 242 kb) [file 424_2012_1132_MOESM2_ESM.jpg]

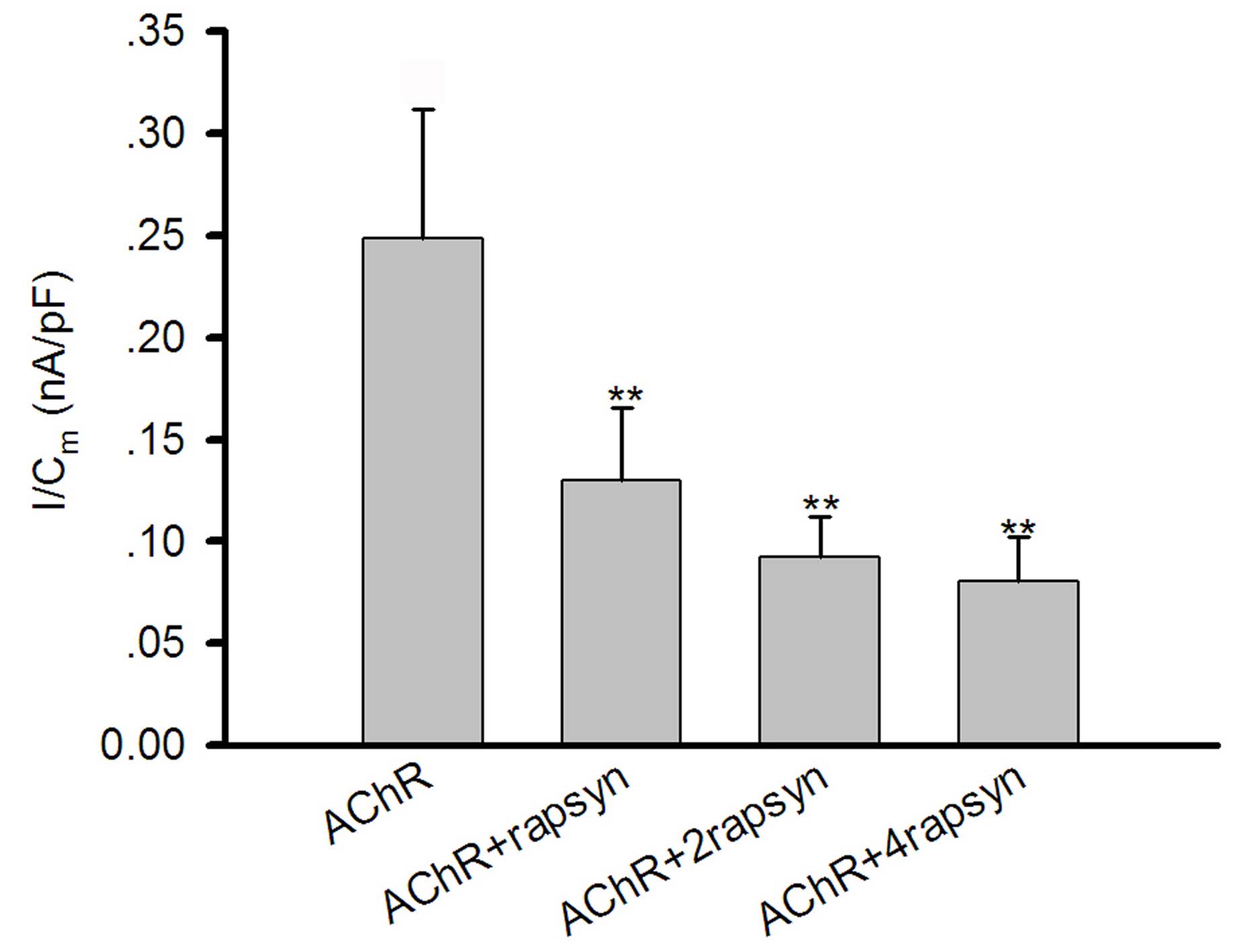

Supplement: Supplementary file 3 — Effect of rapsyn on AChR current density in HEK293T cells. AChRs were co-expressed with rapsyn at cDNA ratios of 1:1, 1:2, and 1:4. The current density was calculated by dividing total whole-cell current by membrane capacitance (I/C m). Number of cells recorded: 32 (no rapsyn), 26 (1:1), 28 (1:2), and 26 (1:4). The pipette holding potential was −70 mV. Data are mean ± SEM, **p < 0.01 (JPEG 111 kb) [file 424_2012_1132_MOESM3_ESM.jpg]

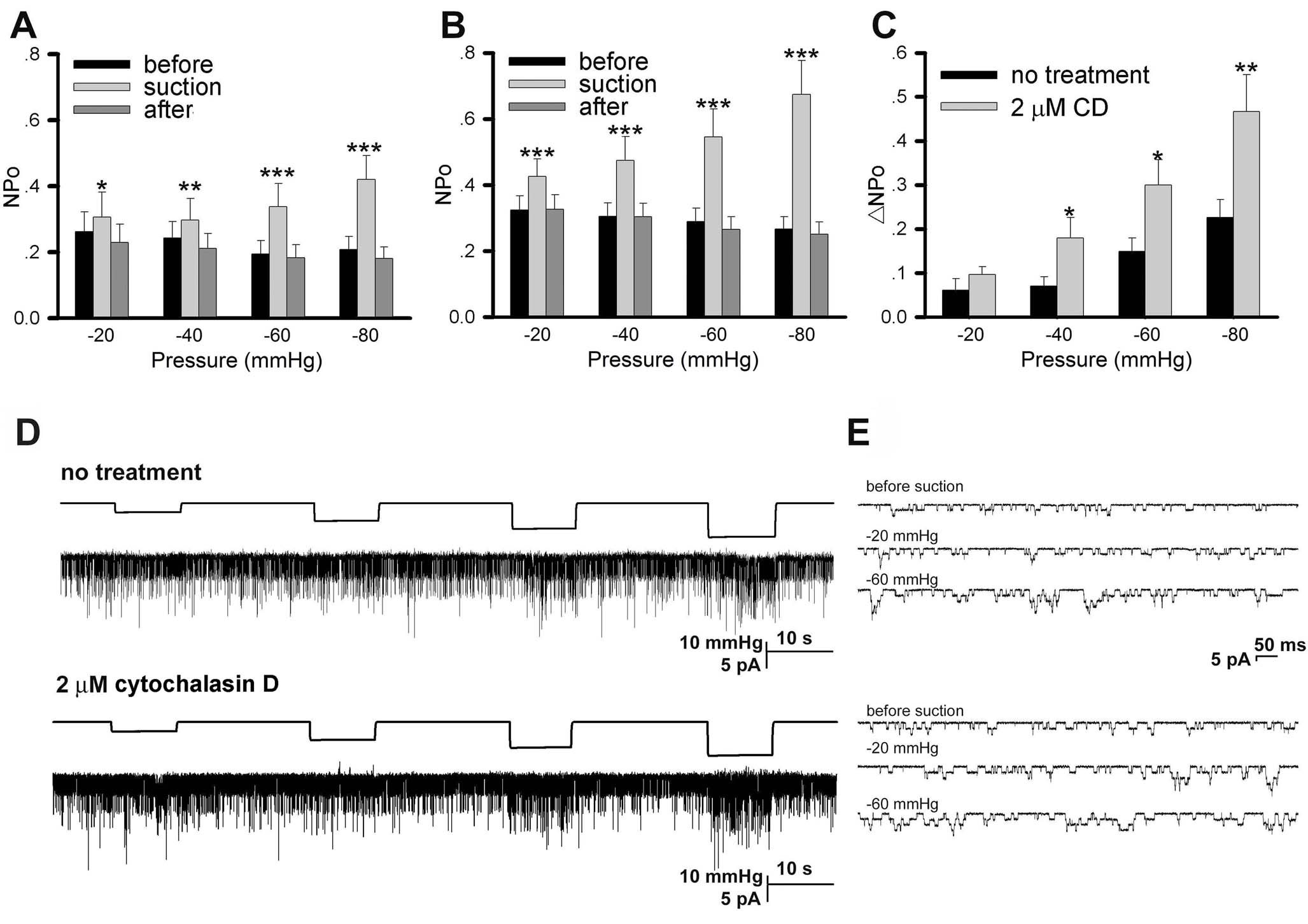

Supplement: Supplementary file 4 — The effect of cytochalasin D on AChR’s mechanosensitivity in C2C12 cells. NPo of control AChR single-channel currents (a) and currents with cytochalasin D treatment (b), before (black), during (gray), and after (dark gray) negative pressure application. ΔNPo comparison between control (black) and cytochalasin D treatment (c). Number of patches n = 20 (control) and 21 (cytochalasin D). Myotubes were pre-incubated with 2 μM cytochalasin D for 30 min for the latter. Sample current traces in two time scales (d, e). Recording pipette contained 0.5 μM ACh. Data are mean ± SEM. *p < 0.05; **p < 0.01; ***p < 0.001 (JPEG 246 kb) [file 424_2012_1132_MOESM4_ESM.jpg]

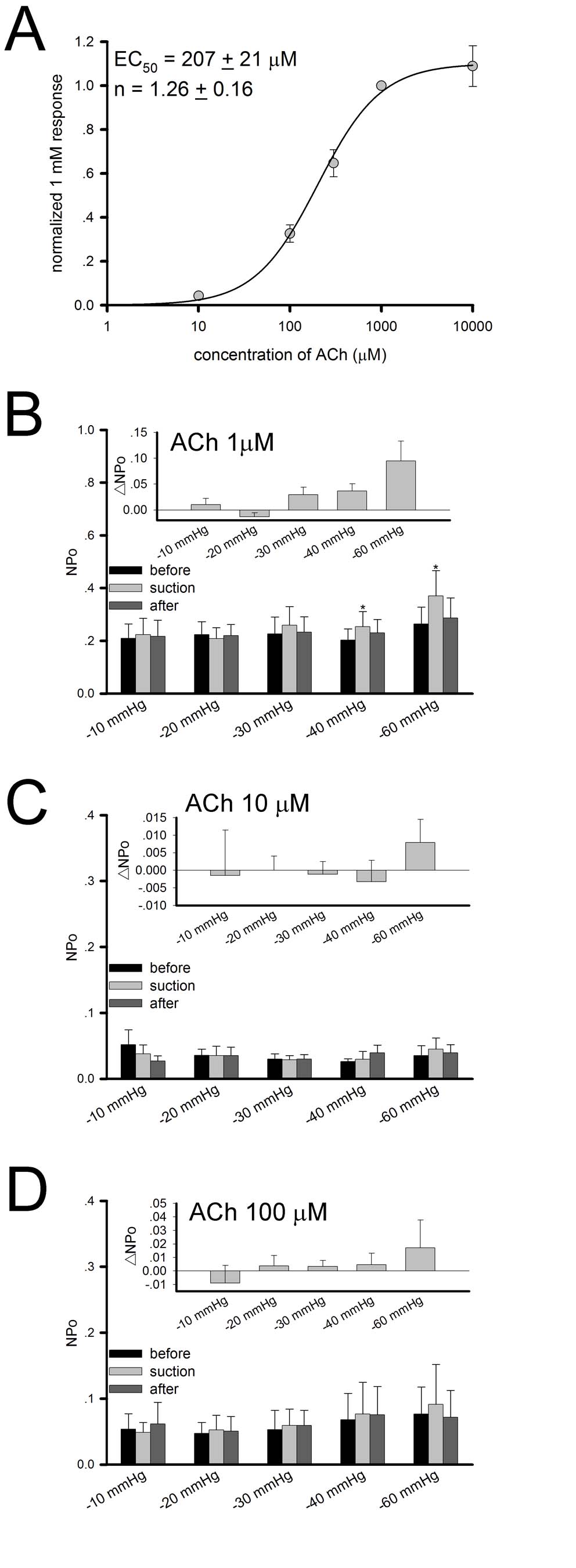

Supplement: Supplementary file 5 — Response of AChRs in rat cortical neurons. a Dose–response of ACh-induced currents in cortical neurons under whole-cell recording mode. b–d Single-channel recordings of ACh-induced currents under negative pipette pressure at three ACh concentrations. In comparison to muscle nAChRs, the neuronal receptors showed negligible mechanosensitivity (JPEG 180 kb) [file 424_2012_1132_MOESM5_ESM.jpg]
